# Label modification and bootstrapping for zero-shot cross-lingual hate speech detection

**DOI:** 10.1007/s10579-023-09637-4

**Published:** 2023-02-18

**Authors:** Irina Bigoulaeva, Viktor Hangya, Iryna Gurevych, Alexander Fraser

**Affiliations:** 1 Ubiquitous Knowledge Processing Lab (UKP Lab), Department of Computer Science, Technical University of Darmstadt, Darmstadt, Germany; 2 Center for Information and Language Processing, LMU Munich, Munich, Germany

**Keywords:** Hate speech, Cross-lingual transfer learning, Class imbalance, BERT, CNN, LSTM

## Abstract

The goal of hate speech detection is to filter negative online content aiming at certain groups of people. Due to the easy accessibility and multilinguality of social media platforms, it is crucial to protect everyone which requires building hate speech detection systems for a wide range of languages. However, the available labeled hate speech datasets are limited, making it difficult to build systems for many languages. In this paper we focus on cross-lingual transfer learning to support hate speech detection in low-resource languages, while highlighting label issues across application scenarios, such as inconsistent label sets of corpora or differing hate speech definitions, which hinder the application of such methods. We leverage cross-lingual word embeddings to train our neural network systems on the source language and apply them to the target language, which lacks labeled examples, and show that good performance can be achieved. We then incorporate unlabeled target language data for further model improvements by bootstrapping labels using an ensemble of different model architectures. Furthermore, we investigate the issue of label imbalance in hate speech datasets, since the high ratio of non-hate examples compared to hate examples often leads to low model performance. We test simple data undersampling and oversampling techniques and show their effectiveness.

## Introduction

Due to the increased digitization of society, the impact of online discourse on everyday life is becoming more pronounced. A single hateful message shared on social media now has the potential to incite violent offline movements, as well as exert a negative emotional impact on millions of readers. For this reason, platforms such as Twitter and Facebook have created community policies to ensure civil conduct on the part of their users. The goal is to filter hate speech, which unlike mere offensive or vulgar content, is exclusively designed to attack or denigrate entire groups of people and has a damaging effect on communities. But with the sheer amount of posts being published, it is becoming difficult for humans to moderate them in a complete and timely manner. Different moderators are also not guaranteed to agree on every decision, even in the presence of well-defined classification guidelines. Moreover, due to their repeated and prolonged exposure to negative content, many moderators experience a decline in mental health Vidgen and Derczynski (2020). For these reasons, automatic hate speech detection has become a field of high interest.

In general, the task of classifying hate speech has been acknowledged as difficult de Gibert et al. (2018). One reason is data scarcity: there are currently few public hate speech datasets available, and the majority of them are for English. Thus, building systems for lower-resource languages is even more challenging Vidgen and Derczynski (2020). An additional difficulty of the task is the need to precisely define hate speech. While many people have an intuitive understanding of what hate speech is, this does not easily translate to a finite set of characteristics that can be used as annotation guidelines. Additionally, many hate speech datasets deal with specific hate speech subtypes, such as hate speech only against refugees, women or certain nationalities, which leads to stark differences between the content of their hate speech classes and making the available resources for a given set of hate speech subtypes in a low-resource language even scarcer.

It is therefore our aim to examine a cross-lingual setup, in which available hate speech resources from a higher-resource language are exploited. We address data scarcity in German, a generally high-resource language but a language for which there are not yet many hate speech datasets available (only a small number of datasets are available compared to English most of which differ in their label sets Vidgen and Derczynski (2020)). Our method is applied in a zero-shot setup that assumes no annotated training data in German. We develop a cross-lingual transfer learning approach based on cross-lingual word embeddings (CLWEs) and neural classifiers to provide access to hate speech data in English. We rely on a widely-used English dataset de Gibert et al. (2018) as our source-language data and the German dataset of the 2018 GermEval Shared Task on the Identification of Offensive Language Ruppenhofer et al. (2018) as our target language data in our experiments. As is often the case with hate speech datasets, the annotation schemas of these two datasets do not fully correspond. Therefore, as we discuss later, we modify their annotation using a few simple rules to ensure label compatibility.

In addition to training only on English, we leverage further data to improve our systems. Towards this end, we bootstrap on two unlabeled German datasets, one of which we crawled from the web. Using an ensemble of our cross-lingual models we predict the labels of previously-unseen data and assign labels with majority voting. We then use this bootstrapped data to further fine-tune the English-trained models. We find that for the majority of our architectures, cross-lingual performance after fine-tuning improves scores within the hate speech class as well as macro-average scores.

Since the majority of social media content is non-hateful, the datasets’ label distributions are skewed towards the no-hate label. Such class imbalances often lead to training issues, especially in case of small training corpora. For this reason we perform a series of additional experiments to test the impact of class ratio on model performance. We create several over- and undersampled versions of our training sets and compare the models’ performance. Our results suggest that severe class imbalance is indeed a problem, but that the best method to overcome it depends on the dataset size.

In sum, our work contributes by addressing three issues in zero-shot cross-lingual hate speech detection: (1) hate speech definition incompatibilities across resources, (2) data scarcity and (3) class imbalance. Regarding hate speech definition, we select compatible datasets and employ manual label modification. Regarding data scarcity, we pursue a cross-lingual setup in which we use English labeled data only to detect hate speech in German. Furthermore, we show that performance can be improved by leveraging unlabeled German sentences. Regarding class imbalance, we show that the imbalanced distributions of hate speech datasets can be compensated with sampling techniques, but that the optimal technique to use may depend on dataset size.

Similar methods have been applied in other tasks and have been used in other hate speech detection setups; however, to the best of our knowledge, no works on hate speech detection apply these methods in a zero-shot, cross-lingual setting.

## Previous work

In this section we give an overview of previous work that addresses the three aforementioned issues of hate speech definition, data scarcity, and class imbalance.

### Hate speech definitions

For as long as hate speech detection has been an area of interest, a multitude of terminologies have been associated with it. Schmidt and Wiegand (2017) note that the earliest work on the phenomenon did not use the term “hate speech” at all, but rather “abusive”, “hostile”, and “flames”. However, despite the vast amount of work that has since been done on detecting hate speech, the term still lacks a universally-accepted definition. In particular, Davidson et al. (2017) observe that the concept of “hate” was previously often conflated with the concept of “offensiveness”, and though more recent works tend to treat hate speech as a subtype of generally-offensive language Wiegand et al. (2018b); Gröndahl et al. (2018); Zampieri et al. (2019), ambiguities and inconsistencies regarding terminology use are still prevalent. The three datasets of HASOC Majumder et al. (2019) distinguish between the categories “Hate Speech” and “Offensive”, the difference being that the former is directed against a group while the latter is directed against an individual. On the other hand, the GermEval2018 dataset of Wiegand et al. (2018b) employs a hierarchical taxonomy, where the label “Offensive” is used as an umbrella term that includes “Abuse”, which is characterized as a “particularly strong form of offensive language” and bears resemblance to the concept of hate speech. Waseem et al. (2017b) also use the term “abuse” rather than “hate speech” in their analysis of contemporary datasets, and underscore the importance of distinguishing the target of abuse, as well as whether the abuse is implicit or explicit. This inspired the OLID taxonomy of Zampieri et al. (2019), which likewise does not use the term “hate speech” as a category label. Instead, the OLID dataset uses the label “Offensive”, which was likened to the “Offensive” category found in the GermEval2018 dataset Wiegand et al. (2018b). However, while the authors of OLID use the term “abuse” in their discussion, and the GermEval dataset contains a category named “Abuse”, these two terms are not implied to have similar meanings. Rather, the term “abuse” in the discussion of Zampieri et al. (2019) is meant to correspond to the label of “Offensive” in their dataset, which in the GermEval2018 dataset would include the label “Abuse” as a subset. Regarding the term “hate speech”, although Zampieri et al. (2019) do not use it as a category label, they nevertheless note that the concept fits into their three-level taxonomy as speech that is (1) offensive, (2) a targeted insult, and (3) targeted against a group.

From these datasets alone, it is clear that there are significant nuances and inconsistencies regarding the use of hate-related terminology. In addition, there are many other terms that are employed in connection to hate speech detection, oftentimes in the context of related, but separate tasks. Fortuna and Nunes (2018) offer a comparison of nine such terms, such as “cyberbullying”, “discrimination”, “flaming”, “toxic language”, and “abusive language”, with explanations of how these concepts differ from the concept of hate speech itself.

Hate speech datasets also differ in annotation schema, which is shown in recent surveys Vidgen and Derczynski (2020); Poletto et al. (2021); Pamungkas et al. (2021a). This variety is due to the multifaceted nature of hate speech, as it can be directed against individuals or groups, be implicit or explicit, and have varying themes such as race, gender, or disability. Quite often, it is seen as advantageous to focus on classifying finer-grained categories than to attempt a binary classification task, where there might be too much variation Poletto et al. (2021). There are datasets whose annotation schemas distinguish between racism and sexism, as well as datasets specific to certain target groups. The HatEval dataset Basile et al. (2019) gathers 13,000 English and 6600 Spanish tweets where the targets of hate speech are either immigrants or women. All tweets with the label “Hateful” must have one of these two targets. The dataset of Bretschneider and Peters (2017) views hate speech as “offensive statements” that express “fear and aggression”, and collects statements of this nature that are directed against foreigners. Meanwhile, hate speech exclusively against refugees and Muslims is the focus of Ross et al. (2016). The dataset of Davidson et al. (2017) defines hate speech as a statement that “expresses hatred towards a targeted group or is intended to be derogatory, to humiliate or to insult members of the group”. The three datasets of HASOC Majumder et al. (2019) do not focus on one particular target and contain a diverse set of sentences labeled as “Hate Speech”. The previously-mentioned OLID dataset of Zampieri et al. (2019) employs a multi-tiered annotation schema that distinguishes on one level whether or not a tweet is “Offensive”, then the type of offensiveness it contains, and finally the target of offensiveness.

Tables 1 illustrates the differences in the taxonomies of various datasets and the contradictory annotations that can arise as a result.^1^ First are sentences 1 and 2, which both direct vulgar language at female politicians. However, Sentence 1 was given the label “Hateful” in accordance with the annotation principles of the HatEval dataset, while Sentence 2 from the dataset of Ross et al. (2016) was given a binary “No” label that signifies the absence of hate speech. Sentences 3 and 4 both direct insults against individuals, however Sentence 3 was annotated as “Hate Speech”, while Sentence 4 was not considered to be hate speech. Sentences 5 and 6 both make statements against the media, which is also a group of people. However, while the GermEval dataset’s label for such a sentence is “Abuse”, the Stormfront dataset labels such a sentence as “Hate”.

**Table 1:** Sentences of similar type carrying different class labels in different datasets. Label names are given as occurred in the datasets. German examples are translated to the best of the authors’ ability

	Sentence	Label	Dataset
1.	@CNN Hillary Clinton does give a shit about women that’s why she lied again she is now keeping all the Harvey Weinstein money GREEDY BITCH	Hateful	HatEval
2.	#Merkel Wie ist diese Schlampe eigentlich auf die Idee gekommen das Land so tiefgreifend zu verändern? #Merkelmussweg #refugeesnotwelcome *en.#Merkel How did this slut ever get the idea to change the country so radically?* * #Merkelmustgo #refugeesnotwelcome*	No (non-hateful)	Ross
3.	@TheErniie You’re a little bitch	Hate	Davidson
4.	You prove that multiculturalism works, your the one that started this troll, thread , fag boy	Nohate	Stormfront
5.	Deutsche Medien, Halbwahrheiten und einseitige Betrachtung, wie bei allen vom Staat finanzierten “billigen” Propagandainstitutionen :P *en. German media, half-truths and one-sided coverage, like with all the “cheap” state-funded propaganda institutions :P*	Abuse	GermEval’18
6.	So you use the jew run media brainwashing service to give credit to soo few black people?	Hate	Stormfront

These distinctions in category assignment are not just observable to the human reader – they also have an impact on model learning. Gröndahl et al. (2018) provide evidence that such blurred distinctions among hate-related categories hinders a model’s ability to generalize to other datasets, regardless of architecture. They observe that nearly all models in their experiments classify non-offensive speech containing vulgar language as hate speech. This underscores the importance of the role of the dataset in the success of a hate speech detection system.

While the aforementioned works have either argued for a unified hate speech taxonomy or proposed their own definitions, there has been a lack of works focusing on mitigating the effects of incompatible taxonomies in the zero-shot cross-lingual setup. Our work aims to close this gap.

### Hate speech data scarcity and cross-lingual transfer

Not only does the content of hate speech datasets pose a challenge, but also the quantity of available datasets, particularly for non-English languages. A comprehensive online catalogue published by Vidgen and Derczynski (2020) shows that, although a large number of languages are represented in hate speech datasets, most datasets are still in English.^2^. Considering the above discussed variance issues of hate speech definition and label sets, multilingual hate speech detection remains an important and relevant task, since social media platforms are multilingual spaces where people may easily communicate in their native tongue Pamungkas et al. (2021a). Due to the costliness of collecting and annotating new data, it is relevant to consider ways of exploiting resources that are already available. As with many low-resource NLP tasks, a common method for achieving good performance is to leverage data from higher-resource languages. This technique is known as cross-lingual transfer learning, and relies on shared representations of languages in order for knowledge in a source language to be transferable to the target language. One form of transfer is machine translation, in which the target language data is automatically translated into the source language before classification. Pamungkas et al. (2021b) use mBERTDevlin et al. (2019) in a training pipeline that utilizes an abusive language lexicon and machine translation. However, translation models require the presence of parallel data to train and may be prone to producing incorrect translations. Therefore, we employ cross-lingual word embeddings, which is a more efficient method of achieving cross-lingual transfer.

Word embeddings provide a means of representing words numerically, thus making important linguistic properties such as semantic similarity accessible to machines. Popular methods are founded upon the idea that semantically-similar words such as “joyful” and “happy” occur in similar contexts Mikolov et al. (2013b); Bojanowski et al. (2017); Devlin et al. (2019). In a cross-lingual NLP task, word embeddings for both the source and target language are needed which are aligned, i.e., the vector of a word in the source language is similar to that of its target-language translation. As a result, a source-language sentence is represented with a similar set of vectors as its translations, thus a model trained on the source language may be applied to the target language without any intermediate steps. Various approaches were proposed to build CLWEs, such as the methods based on the idea of mapping independent monolingual embeddings to a shared vector space Mikolov et al. (2013a); Conneau et al. (2018); Artetxe et al. (2018) or the approaches learning such spaces jointly Devlin et al. (2019). In our work we rely on both types of approaches. More precisely, we use MUSE Conneau et al. (2018) and multilingual BERT Devlin et al. (2019) models.

CNNs, RNNs and transformers are the most commonly-used models for hate speech detection and offensive language detection in general Waseem et al. (2017a); Fišer et al. (2018); Roberts et al. (2019); Ruppenhofer et al. (2018); Struß et al. (2019); Benítez-Andrades et al. (2022); MacAvaney et al. (2019); Pamungkas et al. (2021b); Pamungkas et al. (2021a). With regards to the first two architectures, we examine two setups that achieved good performance on the 2018 GermEval shared task. Xi et al. (2018) used a CNN following Kim (2014), while a combination of CNN and BiLSTMs architectures were used to achieve second-best and best performance in the two subtasks respectively Wiedemann et al. (2018).

Transformer-based architectures such as BERT Devlin et al. (2019) have also been successfully applied to the task. A notable example is the 2019 iteration of the GermEval shared task, where the teams using fine-tuned BERT consistently placed among the top performers Struß et al. (2019). Additionally, several works use BERT in zero-shot cross-lingual setups. Pelicon et al. (2021) and Nozza (2021) use mBERT, the multilingual version of BERT, without any intermediate steps between source-language training and target-language testing. In this work we use mBERT.

Other works propose novel architectures for zero-shot setups. Unlike few-shot setups, where some gold labels in the target language are available, a zero-shot setup does not utilize any labeled target-language data during training. Stappen et al. (2020) propose a novel attention-based method for a zero-shot setup, training on the source language and testing on the target language without any intermediate steps. Jiang and Zubiaga (2021) propose a novel architecture using machine translation as part of their pipeline. Different from these works, we do not use machine translation and we additionally employ data sampling and a bootstrapping step before target-language testing.

Cross-lingual transfer techniques were applied for hate speech detection in Ranasinghe and Zampieri (2020) by training transformer-based architectures on English data and using the learned weights to initialize models which are trained on target language data for improved performance. Similarly, a small number of target language samples were concatenated with the source-language training data in Stappen et al. (2020). In Wiegand et al. (2018a) bilingual word embeddings were used to leverage additional source language data by augmenting the available German training data with English labeled samples. Pamungkas et al. (2021b) use a pipeline that involves an abusive language lexicon and machine translation. Mathur et al. (2018) utilize a cross-lingual transfer procedure for hate speech detection in Hinglish, a code-switched language that uses both Hindi and English words. By first training a CNN and an LSTM on an English dataset, then fine-tuning the models on Hinglish, better performance was achieved compared to a Hinglish-only model. However, this work relied on having labeled data for the target language. In contrast, our approach requires no target language annotations.

Kozareva (2006) present a bootstrapping-based approach that annotates new data for named entity recognition to improve the performance in low-resource scenarios. First a set of classifiers are trained, which are then applied to an unlabeled set with majority voting. The extended corpus is used to improve the performance by retraining the models from scratch. For hate speech detection, Bigoulaeva et al. (2021) combined the bootstrapping procedure of Kozareva (2006) with the fine-tuning procedure of Mathur et al. (2018) by first bootstrapping German-language hate speech data then using it to fine-tune CNN and BiLSTM classifiers. This resulted in improved performance for both architectures. In this work we follow Bigoulaeva et al. (2021), additionally using mBERT alongside the CNN and BiLSTM.

Zia et al. (2022) utilize a similar bootstrapping setup to ours, using the XLM-R model to generate target-language labels and then fine-tuning a monolongual target-language transformer model (either RoBERTa or BERT) on the generated data. Different from them, we use CNNs and LSTMs along with mBERT, and use the artificially-labeled data to fine-tune the same model that produced it.

Equally important to the consideration of model architecture for cross-lingual transfer learning is the choice of datasets. When working with a single hate speech dataset, i.e., the scenario where one annotates datasets for their application needs therefore both training and testing data is provided from the same data source, the problem of compatibility of hate speech definition does not arise. In our cross-lingual setup however, where both a source- and target-language dataset are required, the problem of label inconsistencies surfaces and poses the risk of either poor model performance or too few resources. Depending on the hate speech definition of the target-language requirements, many or all available source-language datasets could be incompatible for use alongside it.

In our experiments, we apply simple rules to make the selected source- and target-language datasets compatible for the cross-lingual evaluation. The idea behind our procedure is the observation that the contents of certain classes can be highly similar across different datasets within the same domain. This observation is present in many previous works. Fortuna et al. (2020) compare the content of six different hate speech datasets to investigate the degree of compatibility between their categories. Using FastText word embeddings to encode semantic similarity, they represent a dataset’s categories as centroid vectors and perform PCA to compare the similarity in relation to the categories of other datasets. They find that many categories across the six datasets are similar in content, despite carrying different names.

In light of this, a viable solution would be to manually merge similar categories into one label. Recent work has shown that this is indeed a reliable and simple method of making certain datasets compatible. Glavaš et al. (2020) assemble a hybrid dataset from three English source datasets that are distinct in domain, with the end goal of creating a multidomain and multilingual (through translation) abusive language resource. In order to ensure dataset compatibility, they manually remap the three-tiered annotation schema of the TRAC dataset Kumar et al. (2018) into the binary annotation schema used by two other datasets: Wulczyn et al. (2017); Gao and Huang (2017). The TRAC dataset features the labels “non-aggressive”, “covertly-aggressive”, and “openly-aggressive”, the latter two of which were relabeled as “abusive” and the former of which was labeled as “non-abusive”. Pamungkas et al. (2021a) also mention dataset relabeling as a common method for cross-lingual hate speech detection and that certain classes may not be combined due to different class definitions. We note that multilingual datasets with compatible annotation across languages were proposed Majumder et al. (2019); Basile et al. (2019); Zampieri et al. (2020), however they do not reflect the real-life scenario where one is required to build a system for a given language that is not present in other datasets. We address this gap by designing our experiments around this setting.

### Class imbalance of hate speech datasets

Making source- and target-language datasets compatible, however, does not address the important issue of class imbalance. Namely, as Vidgen and Derczynski (2020) observe, hate speech is the minority class in most datasets. The dataset of Waseem and Hovy (2016) has been observed to consist of 68% non-hate examples Fortuna et al. (2020). On the one hand, this simulates a real-life scenario, and Pamungkas et al. (2021a) remark that it is important that the class ratio of the test dataset correspond with the training dataset. But on the other hand, an imbalanced class ratio leads to an even smaller amount of available positive examples for the detection of hate speech, and so the models may not learn about hate speech sufficiently.

We explore simple under- and oversampling techniques with various label ratios to show the importance of handling the skewed labeled distribution of hate speech datasets.

Johnson and Khoshgoftaar (2019) differentiate between *data-level* and *algorithm-level* methods for dealing with class imbalance. The former is concerned with influencing the data distribution directly through over- or undersampling the data items. The latter is concerned with adjusting model behavior during training by means of cost-sensitive training, selecting certain loss functions, and altering output thresholds. *Hybrid* methods also exist which combine both data-level and algorithm-level techniques.

Due to their simplicity, we explore over- and undersampling techniques in our work. They respectively involve duplicating random samples from the minority class and removing random samples from the majority class. Previous research with feature-based machine learning models suggests that oversampling delivers slightly better performance than undersampling, likely because undersampling removes data Mohammed et al. (2020); De Smedt and Jaki (2018). We test the efficacy of over- and undersampling hate speech datasets on our neural networks. To our knowledge, there are no other works on zero-shot cross-lingual hate speech detection that investigate the effects of various over- and undersampling ratios.

An additional consideration for oversampling is whether to merely duplicate existing data samples from the minority class, or to generate entirely new samples by automatic means such as SMOTE Chawla et al. (2002). The former method is simpler, but may cause overfitting due to saturating the minority class with similar samples. On the other hand, the latter method generates artificial samples that may not share many common features with the real data.

We performed initial experiments with automatic sample generation using the SMOTE library but found that this resulted in poor performance. Therefore, for oversampling we manually duplicate class samples from the minority class.

## Experimental setup

This section introduces the setup of our experiments. First we discuss our chosen datasets, showing their class distributions and the degree of overlap in hate speech definition. Finally we present our three models based on CNN, BiLSTM, and mBERT architectures respectively.

### Datasets

To ensure the validity of our cross-lingual setup, it was necessary to choose a source- and target-language dataset pair such that the hate speech classes of the two overlapped. Despite English being a high-resource language, choosing a dataset with a narrow-focused hate speech definition would potentially limit the number of German datasets that could be used for testing. For this reason we sought out an English dataset with a broad hate speech definition, since it would more likely be compatible with the available German datasets.

In general, one option for a cross-lingual setup is to use a multilingual dataset that contains both English and German data. One such dataset for hate speech is OLID. However, this is not compatible with our setup, since our aim is to demonstrate what may be done when one needs to find a separate dataset in the source language in order to be able to perform hate speech detection in the target language.

**Table 2:** Sample hate and non-hate comments from the Stormfront dataset de Gibert et al. (2018)

	Sentence	Label
1.	This film tells the story of a poor victimised African boy (Joseph) who was allowed into Ireland	Nohate
2.	There are London areas that could be described as nothing less than little islamic republics	Hate
3.	But unfortunately, Maine’s become the dumping ground for Somalis and other African trash	Hate
4.	It is nothing short of hysterical that you are such a retard you have to TELL us in your username that you are educated	Nohate
5.	Im sorry about being a bitch.i was just hurt.hope you find your talk with someone. ifyouwant to talk to someone who is trying to get their degrees pm me on sf or im me on aol or yahoo	Nohate

One such English dataset is found in de Gibert et al. (2018) who define hate speech as “a deliberate attack directed towards a specific group of people motivated by aspects of the group’s identity”. This dataset features text scraped from the white-nationalist forum Stormfront and will be referred to as the Stormfront dataset. Due to its broad hate speech definition and its decent size (ca. 10,000 examples), it was chosen as the training set for this paper. Table 2 illustrates some ‘Hate’ and ‘noHate’ sentences from the Stormfront dataset. Sentence 1 is not an example of hate speech, since it has a neutral sentiment and does not ascribe the qualities ‘poor’ and ‘victimized’ to an entire group of people. Sentences 2 and 3 are examples of hate speech directed at religious and racial groups, respectively. Sentence 4 is an attack on an individual that uses the derogatory term “retard” to ascribe low intelligence, but was assigned the ‘noHate’ label since it did not address a group. Finally, Sentence 5 uses the profane and derogatory word “bitch” in a non-attacking context.

Our choice for the target dataset was the dataset of German-language tweets presented with the 2018 GermEval Shared Task on the Identification of Offensive Language Ruppenhofer et al. (2018). The shared task focused on the detection of offensive language in general (the coarse-grained task), along with the detection of three of its subtypes (the fine-grained task): ‘Insult’, ‘Profanity’, and ‘Abuse’. Although the dataset does not contain a category that is explicitly designated ‘hate speech’, the category ‘Abuse’ is nevertheless defined in terms that are similar to the hate speech definition of the Stormfront dataset. Namely, a tweet is assigned the ‘Abuse’ label if “... the target of judgment is seen as a representative of a group and it is ascribed negative qualities that are taken to be universal, omnipresent and unchangeable characteristics of the group” Ruppenhofer et al. (2018). Importantly, this definition keeps the nature of the target group general and is therefore compatible with the hate speech definition in de Gibert et al. (2018). We therefore take this category to be the correspondent of the Stormfront dataset’s ‘Hate’ class, despite it carrying the name ‘Abuse’, and use it our test set for the cross-lingual experiments. However, the label scheme of the GermEval dataset nevertheless had to be aligned with that of the Stormfront dataset, which we discuss in Sect. 3.1.1.

**Table 3:** Sample comments from the GermEval dataset Wiegand et al. (2018b)

	Sentence	Label
1.	@ShakRiet @Heinrich_Krug So ist es....wir haben Maria vergessen... als hätte sie nie existiert....schämt euch...! *en. That’s how it is... we have forgotten Maria...as if she never existed...* *shame on you...!*	Other
2.	Martin Schulz ist 2x sitzen geblieben und hat keinen Schulabschluss. Wie kann denn so ein Nulltipper als Kanzlerkandidat aufgestellt werden? *en. Martin Schulz was held back in school twice and has no diploma.* *How can that kind of idiot be held for a chancellor candidate?*	Insult
3.	Wir sollten den deutschen Kinder und Frauen gedenken die durch den #Islam ermordet wurden. *en. We should commemorate the German children and women murdered* *by #Islam*	Abuse
4.	@HenHoffgaard @mboe0407 Da die Kirche jeher den Herrschenden in den Arsch gekrochen ist, inkl. Hitler, wundert es mich nicht *en. Well since the church always kissed the ass of the ruling elite,* *including Hitler, this doesn’t surprise me*	Profanity
5.	@Nacktmagazin @DuHugonotte Und zum Nachtisch einen Mohrenkopf *en. And for dessert a Mohrenkopf (head of a Moor / a kind of candy)*	Abuse

Table 3 shows samples of various classes from the GermEval dataset. Sentence 1 expresses negative emotions about a specific person being forgotten but does not seek to attack or denigrate anyone. Sentence 2 insults a single politician with a nickname “Nulltipper” *(en. “idiot”)* and the lack of a school diploma to ascribe low intelligence. Sentence 3 is an example of “Abuse”, since it ascribes acts of murder to an entire religious group. Sentence 4 is an example of the “Profanity” category as it contains the profane phrase “in den Arsch gekrochen”, while not being verbosely critical or attacking. Finally, Sentence 5 is another example of the “Abuse” class, since it uses the term “Mohrenkopf”, which typically denotes a kind of candy, as a derogatory designation for dark-skinned individuals.

Despite the alignment of annotation categories, domain differences between the source- and target-language datasets may pose challenges to cross-lingual transfer. In our case, the domain of the Stormfront dataset is a message forum and the domain of the GermEval dataset is Twitter. In the former case, messages are often lengthy and can be written in a structured, formal style. In the later case of tweets, the messages have a length limit and are often informal, featuring slang and abbreviations. A prevalence of lengthy and formal messages in the Stormfront dataset might therefore inhibit a model’s performance on the tweet-based target dataset. From manual examination of the Stormfront dataset, however, we found that shorter, informal messages similar to the tweet style were the majority, while essay-like posts were the minority. Additionally, we filter out lengthy posts as explained in Sect. 4.1.2. Although there are some domain differences, it is more important to use datasets with compatible hate speech definitions.

#### Annotation discrepancies

Examining the two datasets’ hate speech definitions and labeled hate speech examples in Tables 2 and 3, it is clear that GermEval’s “Abuse” category corresponds with the ‘Hate’ label of the Stormfront dataset. However, the differing annotation taxonomies as well as the different names attached to the compatible categories pose problems for machine learning models, which will expect consistent annotations between training and testing. Therefore it was necessary to make a few simple adjustments to the datasets before beginning our experiments.

The Stormfront dataset’s distinction between ‘Hate’ and ‘noHate’ is an example of a binary annotation schema. Additionally the dataset contains a ‘Relation’ label for sentences that had to be considered in context with others to acquire a hateful meaning, and a ‘Skip’ label for when the sentence was either non-English or not meaningful enough to be given either of the binary labels. In contrast, the GermEval dataset features a two-tiered annotation schema: each tweet carries a label for the coarse-grained task of ‘Offense’ vs ‘Other’ as well as a fine-grained label that specifies the subtype of offensiveness: either ‘Insult’, ‘Profanity’, or ‘Abuse’.

To ensure compatibility between these two datasets, we made modifications to their labeling schemas that were motivated by the datasets’ specific class definitions. First we simplified the annotation schema of the fine-grained GermEval data into a binary schema. As per the discussion in Sect. 3.1, we took GermEval’s ‘Abuse’ label to be the counterpart of the Stormfront dataset’s ‘Hate’, since the definition of the ‘Abuse’ category was the most compatible with the hate speech definition of de Gibert et al. (2018). Analogously we relabeled the GermEval comments belonging to the ‘Other’, ‘Insult’, and ‘Profanity’ classes as ‘noHate’, since the respective definitions of these categories fail to fulfil one or more aspects of the hate speech definition in de Gibert et al. (2018). An ‘Insult’ in GermEval, for example, is an attack on an individual rather than a group; instances of ‘Profanity’ are never attacks; and instances of ‘Other’ are always non-hateful. Next, we relabeled all ‘Skip’ and ‘Relation’ samples from the Stormfront dataset to conform with the binary schema. The 92 comments that carried the label ‘Skip’, indicating that they were either non-English or not informative, were relabeled as ‘noHate’. The 168 instances of the ‘Relation’ class were relabeled as ‘Hate’, since these sentences were always hateful when placed in context.

After relabeling was completed, we split both datasets into training, development, and test sets. From the Stormfront dataset we form our EN-TEST set by selecting random ‘Hate’ and ‘noHate’ samples, with a class ratio that roughly reflects the data distribution. We kept the size of this dataset small in the interest of preserving resources for training. Next we draw an equal amount of ‘Hate’ and ‘noHate’ samples that did not overlap with EN-TEST for our EN-DEV dataset. The remaining samples formed EN-TRAIN.

For the split-up of our GermEval dataset, we follow the work of Wiedemann et al. (2018). The GermEval shared task data comes with an official train and test dataset, the latter of which we keep and name DE-TEST. For our train/dev split, we transfer the last 809 samples from the provided training set to a new development set named DE-DEV for hyperparameter tuning. The remaining samples formed our DE-TRAIN dataset, which will be used only in the bootstrapping experiments. Table 4 shows the class distribution of the resulting datasets. These will form the basis of our experiments. See Tables 5 and 6 to compare to the original, unmodified versions of the datasets.

**Table 4:** Class distributions of the English and German datasets after relabeling and train/dev splitting

	Nohate	Hate	Ratio (approx.)
EN-TRAIN	9018	1281	7:1
EN-DEV	134	20	7:1
EN-TEST	427	63	7:1
DE-TRAIN	3345	855	4:1
DE-DEV	642	167	4:1
DE-TEST	2759	773	4:1

**Table 5:** Original Stormfront dataset before relabeling and train/dev splitting

	Nohate	Hate	Relation	Skip
Stormfront	9488	1196	168	92

**Table 6:** Original GermEval datasets before relabeling and dev splitting from the training set. These were the datasets provided to the shared task participants

	Other	Abuse	Insult	Prof.
GermEval train	3321	1022	595	71
GermEval test	2330	773	381	48

#### Addressing class imbalance

After the relabeling and train/dev splitting process was complete, we addressed the imbalanced class distributions of the training datasets. Examining Table 4, it is clear that there is a greater abundance of ‘noHate’ compared to ‘Hate’. This reflects the real-life pattern of hate speech occurring less commonly than regular text. But this poses difficulties for machine learning models, which need plenty of data from both classes in order to be able to generalize Madukwe et al. (2020); Vidgen and Derczynski (2020).

Previous research suggests that over- and undersampling the data yields good model performance De Smedt and Jaki (2018), thus we also experiment with these techniques by testing various class balance ratios. Since we found oversampling to a balanced class ratio to be the most effective, we manually duplicate the ‘Hate’ examples from EN-TRAIN to produce EN-OS[1:1]. The balanced 1:1 class ratio represents the best-case scenario where neither class is in the minority. The resulting dataset is shown in Table 7. For more details about our datasampling experiments we refer to Sect. 4.2.

**Table 7:** The unmodified EN-TRAIN dataset and its balanced oversampled version: EN-OS[1:1]

	Nohate	Hate	Ratio (approx.)
EN-TRAIN	9018	1281	7:1
EN-OS[1:1]	9018	9018	1:1

### Models

**Fig. 1:**
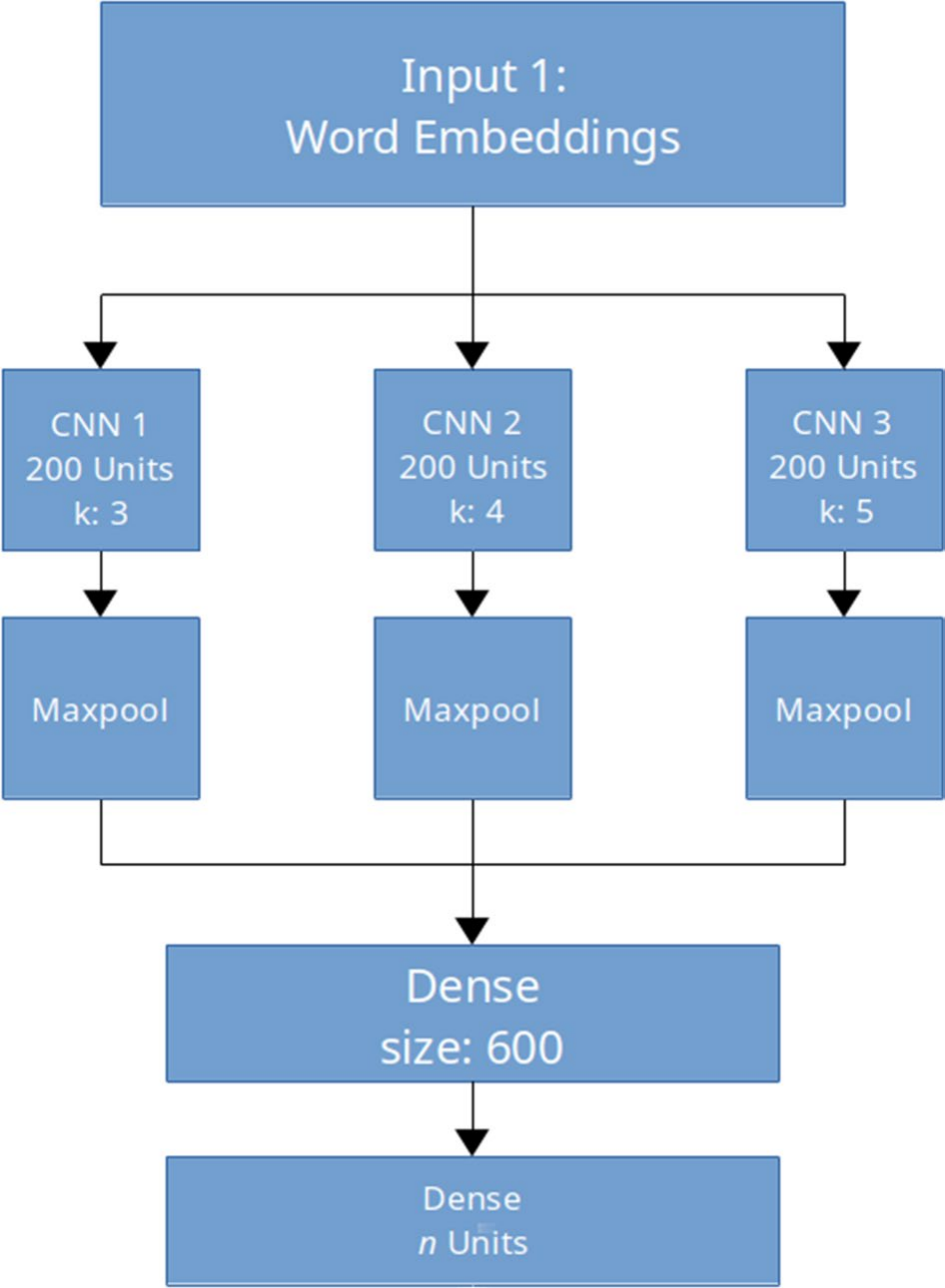
CNN model architecture with multiple convolutional filters with size *k*

**Fig. 2:**
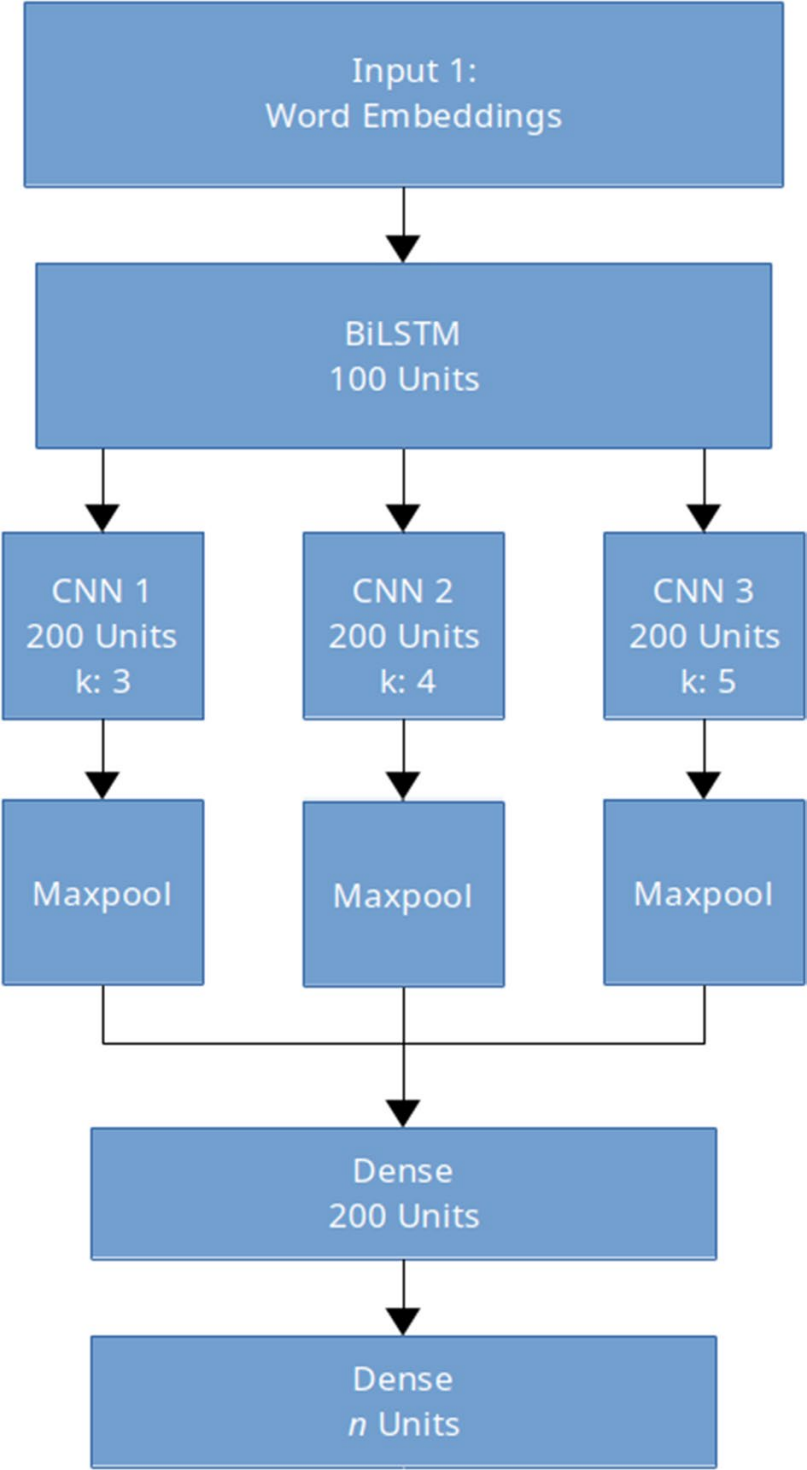
BiLSTM model with convolutional layers on top

In our experiments we focus on evaluating neural network architectures, using monolingual models that have been popularly applied to the task in the past. Our first model is a CNN classifier following Kim (2014) depicted in Fig. 1. This model accepts an embedding layer as an input and feeds it into a convolution layer with a variable number of filters. Global max-pooling is performed on the convolution output, and the result is passed through a dense layer. The input word embeddings can either be randomly-initialized, pre-loaded from an outside source, or fine-tuned during training. We used our pre-trained CLWEs as described below, and did not update them during training. For the remaining model hyperparameters, we used the default values.^3^

To produce our CLWEs, monolingual embeddings were first trained using FastText SkipGram Bojanowski et al. (2017) over English and German NewsCrawl corpora Bojar et al. (2015) which contain text dating from 2007 to 2013 and were preprocessed with Moses tools Koehn et al. (2007). The resulting embeddings were mapped with MUSE Conneau et al. (2018). We used the default parameters of the above mentioned tools.

Our second model is based on the neural model of one of the participants of the 2018 GermEval Shared Task Wiedemann et al. (2018), with some modifications for compatibility with our cross-lingual setup. In our version as shown in Fig. 2, an input layer of our CLWEs was fed into a BiLSTM layer of 100 units. The output of this BiLSTM layer was then fed into a convolution layer with three feature maps of 200 units each, with respective kernel sizes of 3, 4, and 5. Global max-pooling was applied after each convolution, and the output of this step was fed to a dense layer of 100 units.

Our third architecture is multilingual BERT, which was pre-trained on Wikipedia data from 104 languages Devlin et al. (2019). This architecture has the advantage of not needing CLWEs as a resource and can be tuned and tested on a source and target language directly. For the sake of consistency in discussions about the other two architectures, we will henceforth refer to the process of tuning mBERT as “training”.

## Results

We conduct our cross-lingual experiments by training the three architectures from Sect. 3.2 on English and testing on German. We use our EN-OS[1:1] dataset for training. Since the testing language was German, hyperparameters such as epoch count, learning rate, and class weights were optimized on DE-DEV. In addition to the per-class scores, we calculate the macro-average F1 score, as this metric was used by the GermEval shared task.

Table 8 shows the performance of these models when tested on DE-TEST. All three models manage to transfer their knowledge of ‘noHate’ from English to German, with the CNN and mBERT in particular achieving classwise ‘Hate’ scores greater than 50 points: 67.44 recall for the CNN and 52.29 precision for mBERT, respectively. Scores were significantly higher in the ‘noHate’ class: The CNN achieved 78.82 precision and mBERT achieved scores above 60. The BiLSTM had the highest performance in ‘noHate’, with precision, recall, and F1 scores all above 75. This is notable since we did not use German-language data at any point. The macro-average scores of the CNN and BiLSTM were relatively tied, however the BiLSTM achieved a macro-average F1 score that was nearly as high as that of mBERT. mBERT’s macro-average scores were the highest among the three models. These results show that cross-lingual training with neural networks is a viable option even when no target-language data is available. These three models will form the ensemble used in Sect. 4.1.

Table 9 shows the hyperparameters that gave optimal performance on EN-OS[1:1]. We observed that mBERT preferred small batch sizes, its scores slightly dropping as batch size was increased. The CNN and BiLSTM in contrast preferred much larger batch sizes and learning rates and exhibited poorer performance when the batch size was lowered. Class weight ratios implemented into the loss function were a relevant parameter for the CNN, which required a slightly greater weight for the ‘noHate’ class. Despite this measure the CNN exhibited severe overfitting behavior, becoming skewed towards predicting only one of the two class labels, which is why it achieved higher ‘Hate’ F1 score (with high recall and low precision) but lower ‘noHate’ score compared to the other two models. Notably this pattern persisted despite class weight and learning rate tuning. Training on a single epoch with a large batch size yielded optimal performance. We refer to the bootstrapping experiments in the following section for further discussion about the CNN performance.

**Table 8:** Model performance on DE-TEST after training on EN-OS[1:1]

Model	Accuracy	Nohate			Hate			Macro-Avg		
		P	R	F1	P	R	F1	P	R	F1
CNN	40.91	78.82	33.56	47.07	21.94	67.44	33.11	50.38	50.50	40.09
BiLSTM	70.44	77.80	86.99	82.14	19.69	11.38	14.43	48.74	49.19	48.28
mBERT	66.39	67.83	93.30	78.55	52.29	14.23	22.37	60.06	53.77	50.46

**Table 9:** Optimal hyperparameters for training on EN-OS[1:1]. The first two columns represent class weights, which were not implemented for mBERT

	Nohate	Hate	Dropout	Learn rate	Batch sze	Epochs
CNN	0.6	0.4	0.7	10^−4^	50	1
BiLSTM	0.5	0.5	0.2	30^−3^	40	30
mBERT	–	–	0.2	10^−5^	5	10

### Bootstrapping

Although cross-lingual transfer learning techniques are applicable to zero-shot hate speech detection, the discussed data scarcity issues, such as low amount of positive hate speech labeled examples, hinder the performance. To mitigate these issues, this phase of cross-lingual experiments is centered around data augmentation and fine-tuning. For this we relied on two target language unlabeled datasets which we labeled automatically using an ensemble-based approach following Bigoulaeva et al. (2021). Our relabeling ensemble consisted of the three neural models in Table 8. We test these models on two sources of German data: the DE-TRAIN dataset (See Table 4) and the DE-NEW dataset to be detailed in Sect. 4.1.2. For each of the two datasets, we applied all three of our models and assigned a final label to each sentence based on majority voting.

For each bootstrapping dataset we take the three models from Table 8, which had originally been trained on EN-OS[1:1], and resumed their training on the bootstrapping dataset, using altered hyperparameter settings as needed to optimize performance. We then test the performance of the fine-tuned models on DE-TEST.

#### Bootstrapping on DE-TRAIN

In this first phase of the bootstrapping experiments, we apply our ensemble to the DE-TRAIN dataset and collect the majority-vote classification results into a new dataset called DE-REL*. We simulate DE-TRAIN as an unlabeled dataset, since it was not used for training of our models.

Table 10 shows the confusion matrix for the labels of DE-REL*. It is clear that this dataset consists predominantly of ‘noHate’ examples, with a severely imbalanced ratio of 43:1. 573 true ‘Hate’ examples were mistakenly labeled by the ensemble as ‘noHate’, while 42 true ‘noHate’ examples were mistakenly labeled as ‘Hate’. Proportionally more classification errors were made in the ‘Hate’ class, reflecting the models’ higher precision, recall, and F1 scores in ‘noHate’ as can be seen from Table 8.

The Labels of DE-REL*

**Table 10:** Confusion matrix of the ensemble-relabeled DE-REL* compared to the original annotations in DE-TRAIN. Gold and predicted labels are shown in the rows and columns respectively

	Nohate	Hate
Nohate	2688	42
Hate	573	34
Total	3261	76

Table 14 provides a closer look at some correctly- and incorrectly-classified examples from DE-REL*, as compared to the original gold labels of DE-TRAIN. Sentence 1 was correctly labeled by the ensemble as ‘Hate’, as it attributes negative qualities such as violence to a religious group. Sentence 2 was also correctly classified as hate speech, as it expresses approval of prejudiced actions towards people with brown skin. Sentence 3 was correctly recognized as ‘noHate’, although it contains a potentially contentious word ‘Hetze’ (*en. ‘hate, agitation’*), which often occurs in contexts of hate speech. This indicates that the ensemble has some knowledge of hate speech features that go beyond lexical cues. Finally, Sentence 4 was falsely labeled by the ensemble as ‘noHate’. This was likely a challenging example for the ensemble due to it being a form of gender-related hate speech that is not abundantly encountered on a white supremacy forum.

***Performance***

Table 11 shows the English-trained models’ performance on DE-TEST after fine-tuning on DE-REL*. Both mBERT and the BiLSTM improve their performance in several areas. The BiLSTM’s classwise recall and F1 for ‘Hate’ increased by 4.14 points and 3.04 points, respectively. Its macro-average F1 increased by 0.49. mBERT’s classwise ‘Hate’ improvements were more modest, its precision increasing by 1.56 points and its F1 by 1.27. Additionally its macro-average F1 increased by 0.71. The BiLSTM’s greater improvements could be due to the model having had too little training data before, while mBERT had already become mostly saturated by the English training data.

The only model to perform worse after fine-tuning was the CNN, which during training outputted either only ‘Hate’ or only ‘noHate’ predictions. The latter is associated with higher macro-average performance since ‘noHate’ is the majority class of DE-TEST. This result is likely due to poor initial training of the CNN. Recalling from Sect. 4, the CNN was trained on EN-OS[1:1] for only one epoch, as it exhibited overfitting behavior otherwise. We included this model in our bootstrapping ensemble, as its sufficiently-varied predictions on DE-TEST after training on EN-OS[1:1] initially suggested that the model was not broken. It is likely however that this initial training was suboptimal and that the single epoch of training was not enough for the CNN to sufficiently learn from its training data.

**Table 11:** Model performance on DE-TEST after training on EN-OS and fine-tuning on DE-REL*

Model	Accuracy	Nohate			Hate			Macro-Avg		
		P	R	F1	P	R	F1	P	R	F1
CNN	78.11	78.11	100.00	87.71	0.00	0.00	0.00	39.06	50.00	43.86
BiLSTM	67.89	77.72	82.57	80.07	19.97	15.52	17.47	48.84	49.05	48.77
mBERT	66.70	68.07	93.30	78.71	53.85	15.14	23.64	60.96	54.22	51.17

Table 12 shows the hyperparameter settings that were used for fine-tuning on DE-REL*. We observed that tuning the class weights for the CNN as well as the dropout had no effect on the overfitting performance. The BiLSTM however achieved balanced performance with similar hyperparameter settings to those of the CNN. mBERT preferred a smaller batch size and improved performance when fine-tuned for more epochs than the other architectures.

Regarding our CNN, the likely reason for its poor fine-tuning performance is poor initial training on EN-OS[1:1]. Since EN-OS[1:1] is balanced, and the CNN’s scores on DE-TEST in Table 8 were comparable to the other models, a likely conclusion is that its poor fine-tuning performance was caused by the bootstrapping datasets. Recent works have suggested that fine-tuning models on the bootstrapping labels they themselves produced can amplify these models’ preexisting biases towards certain labels Wei et al. (2021); Wang et al. (2022). A direction for further investigations would be to explore this angle, including why the BiLSTM and mBERT architectures were less susceptible to becoming biased during fine-tuning.

**Table 12:** Optimal hyperparameters for fine-tuning on DE-REL*. The first two columns represent class weights, which were not implemented for mBERT

	Nohate	Hate	Dropout	Learn rate	Batch size	Epochs
CNN	0.01	0.99	0.2	$10^{-6}$	30	1
BiLSTM	0.1	0.9	0.7	$10^{-6}$	50	2
mBERT	–	–	0.5	$10^{-5}$	10	10

#### Bootstrapping on German stormfront data

In the second bootstrapping experiment, we use the DE-NEW dataset collected by Bigoulaeva et al. (2021), which was crawled from a German-language thread within the Stormfront forum.^4^ This dataset was originally collected for a zero-shot transfer learning experiment, therefore there was no annotation process conducted to assign gold labels to the data samples. Since we likewise deal with a zero-shot setup in this work, we do not annotate DE-NEW with gold labels.

At the time of crawling, the source thread had around 5500 posts^5^. These consisted predominantly of comments written in German, although many were written in English. To account for the typical prevalence of lengthy posts in a forum setting, Bigoulaeva et al. (2021) considered each paragraph distinguished by a newline to be a separate text sample. Before the data could be used for training, some manual preprocessing was performed to ensure compatibility with the format of a tweet. Table 13 shows what texts were kept and removed. Additionally, the following errors in the texts were manually corrected and kept:

- ‘tut mir’ and ‘leid’ $\rightarrow$ ‘tut mir leid’
- ‘d aß’ $$\rightarrow $$ ‘daß’

**Table 13:** Preprocessing steps for the DE-NEW dataset

	Removed		Kept
1.	Non-German text	1.	Quotes or news article snippets under 1000 characters.
2.	Bullet-point lists		
3.	Quotes from books, articles, etc. over 1000 characters	2.	Mixed English/German sentences
4.	Extremely short lines: names, one-word responses, timestamps, letter salutations	3.	Multi-line interview dialogue, with each line considered as a distinct text sample.
5.	Lines or sentences that were cut off without any clear continuation	4.	Mixed English/German sentences and Anglicisms

**Table 14:** Correct and incorrect ensemble labels for either DE-REL* or DE-NEW. Gold labels for DE-NEW are given by the authors in brackets

		Sentence	Ensemble	Gold
DE-REL*	1.	#Islamisierung ”Zusammenstöße zw GLÄUBIGEN (Richtig: #Islamisten) & Sicherheitskräften” - #Tagesschau sendet bereits im #Scharia-Modus *en. “Confrontations between BELIEVERS (Actually: #Islamists) & the police force” - #Tagesschau is already broadcasting in #Sharia-Mode*	Hate	Hate
	2.	Dortmund haut wieder auf die Kacke ! Die wollen die ganzen Braunhäutigen verjagen mit dem Slogan BUNT STATT BRAUN !! BRAVO !!! *en. Dortmund is rising up again! They want to drive out all the brown-skinned with the slogan “COLORFUL INSTEAD OF BROWN!” BRAVO!*	Hate	Hate
	3.	@Dora_Bromberger @lawyerberlin Hetze haben weder Fakten noch Argumente je ersetzt. *en. Hate has never replaced facts or arguments.*	Nohate	Nohate
	4.	@Sammy_aus_Cux Von mir aus gibt es dieses intersexuell aber die eigentlichen Geschlechter sind männlein und weiblein *en. For all I care these intersexuals do exist but the real sexes are male and female*	Nohate	Hate
DE-NEW	5.	Das Problem mit diesen Mischlingsehen ist, dass diese Maenner sich nicht um Ihre Familie kuemmern und die meisten dann von Sozialhilfe leben. Die Kinder sehen aus wie Orang-Utans, sind nicht sehr intelligent und werden ueberall gehaenselt, was wiederum zu einem kriminellen Lenbenstil fuehrt. *en. The problem with these mixed marriages is that these men don’t care for their families and most of them then live off of welfare. The kids look like orangutans, aren’t very intelligent, and get teased everywhere, which then leads to a criminal lifestyle.*	Hate	(Hate)
	6.	Dreckspack verfluchtes! Wie bescheuert kann man sein? *en. Cursed pack of scum! How crackbrained can you get?*	Hate	(Hate)
	7.	Ich glaube die Türken warten auf die Ergebnisse der türkischen Experten *en. I think the Turks are waiting for the results of the Turkish experts.*	Nohate	(Nohate)
	8.	Der in Tutzing in Oberbayern lebende kanadische **Holocaust-Leugner** Alfred Schaefer ist wegen Volksverhetzung angeklagt. Der 63-Jährige selbst hat den Verhandlungstermin am 4. Mai vor dem Amtsgericht Dresden mit den Worten er sei “vor die Inquisition geladen” publik gemacht und angekündigt, den Prozess dazu zu nutzen, in langatmiger Form den **nationalsozialistischen Völkermord** an den Juden in Frage zu stellen. *en. The Canadian* ***Holocaust-denier*** *Alfred Schaefer, who lives in Tutzing in Upper Bavaria, has been charged with sedition. On his part, the 63-year-old made his trial appointment before the court of Dresden on the 4th of May public by saying he had been “invited by the Inquisition”, and announced that he would use the process to verbosely call into question the* ***national-socialist genocide*** *of the Jewish people.*	Nohate	(Nohate)
	9.	Könnte es sein, jene wenigen Privilegierten beginnen zu begreifen, daß Macht und Reichtum nichts gegen das Streben nach historischer Exaktheit ausrichten können? *en. Could it be that those few privileged are beginning to realize that power and riches have nothing against the striving towards historical precision?*	Hate	(Nohate)

As a result of this preprocessing, DE-NEW contains 6,586 text samples, all or nearly all written in German. This dataset was used as the training set during fine-tuning.

Table 15 shows the class distribution of DE-NEW compared to DE-REL*. DE-NEW is the larger, but interestingly the ensemble’s relabeling resulted in both datasets having similar class ratios. This could indicate that the stylistic differences between the Twitter-based text of DE-REL* and the forum-based text of DE-NEW were not a hindering factor for the ensemble.

**Table 15:** Class distributions of the two bootstrapped datasets

	Nohate	Hate	Ratio (approx.)
DE-NEW	6437	142	45:1
DE-REL*	3261	76	43:1

***The Labels of DE-NEW***

Since we had no gold labels of DE-NEW to evaluate our ensemble’s classifications, we manually examined several examples and judged them strictly according to the points of the hate speech definition in de Gibert et al. (2018). Table 14 shows five classifications made by the ensemble.

Sentence 5 was correctly identified as ‘Hate’, as it is derogatory towards families of mixed races, employing dehumanizing comparisons and attributing low intelligence. Sentence 6 was also correctly identified as ‘Hate’. Although the target group is unclear, the group is also described with dehumanizing language and is portrayed as being dirty and unintelligent. Sentence 7 is a neutral descriptive statement that does not attack the group of Turkish people, and was correctly recognized as ‘noHate’. Similarly, Example 8 is a neutral descriptive account, despite discussing a figure of controversy and using terminology (shown in bold) that would likely be associated with hateful discourse: “nationalsozialistischen Völkermord” (*en. National-Socialist/Nazi genocide*), and “Holocaust-Leugner” (*en. Holocaust-denier*). Together with Sentence 7, this again shows that the ensemble learned more complex features of hate speech than lexical cues (See Sect. 4.1.1).

Sentence 9 was another challenge for the ensemble. It was labeled as ‘Hate’ despite not having any telling signs of hate speech, likely due to discourse about privilege, power and riches having occurred elsewhere in the Stormfront data in more hateful contexts. This would lead the models of the ensemble to recognize that these groups are typically ones to be attacked. Nevertheless we judged this sentence to be an example of ‘noHate’, since when the sentence is considered in isolation it does not attack or dehumanize the groups in question.

Performance Table 16 shows the models’ performance on DE-TEST after training on EN-OS and fine-tuning on DE-NEW. As in the first fine-tuning experiment, the BiLSTM and mBERT improved their scores over the original versions trained on EN-OS[1:1]. This time the BiLSTM’s classwise ‘Hate’ scores improved to a lesser degree, with its precision increasing by 0.55 points and its classwise recall and F1 score dropping slightly. Nevertheless this precision value was higher than after fine-tuning on DE-REL*. All three of its macro-average measures improved as well and were also higher than in the first fine-tuning round (See Table 11). mBERT experienced a slight decrease in macro-average and classwise ‘Hate’ scores. The only ‘Hate’ score to improve was precision, which increased by 0.30 points. Classwise recall and F1 in ‘noHate’ increased while the precision decreased.

This lesser degree of improvement in ‘Hate’ compared to the first fine-tuning experiment could have been caused by DE-NEW’s slightly larger ratio of ‘noHate’ to ‘Hate’ as compared to DE-REL* in Table 15.

Additionally, the slight domain difference (see Sect. 3.1) compared to the test data could further explain these results. As in the previous bootstrapping experiments, the CNN model worsened after fine-tuning, likely due to poor initial training.

Table 17 shows the hyperparameter settings that were used for fine-tuning on DE-NEW. As before, tuning these hyperparameters did not mitigate the CNN’s overfitting performance. The BiLSTM improved with a smaller batch size than in the previous fine-tuning experiment as well as with a lower learning rate and higher dropout. mBERT’s improvements in this fine-tuning experiment were also correlated with different hyperparameters, in this case a small batch size, a lower learning rate, and a reduced epoch count. The reason for this behavior could be the differing class ratios between DE-REL* and DE-NEW.

**Table 16:** Model performance on DE-TEST after training on EN-OS[1:1] and fine-tuning on DE-NEW

Model	Accuracy	Nohate			Hate			Macro-Avg		
		P	R	F1	P	R	F1	P	R	F1
CNN	78.11	78.11	100.00	87.71	0.00	0.00	0.00	39.06	50.00	43.86
BiLSTM	71.04	77.89	87.86	82.58	20.24	11.00	14.25	49.07	49.43	48.41
mBERT	66.31	67.27	95.28	78.86	52.59	10.15	17.02	59.93	52.71	47.94

**Table 17:** Optimal hyperparameters for fine-tuning on DE-NEW. The first two columns represent class weights, which were not implemented for mBERT

	Nohate	Hate	Dropout	Learn rate	Batch size	Epochs
CNN	0.01	0.99	0.9	$10^{-4}$	2	1
BiLSTM	0.1	0.9	0.9	$10^{-7}$	20	1
mBERT	–	–	0.6	$10^{-7}$	1	5

### Data sampling experiments

In this section we conduct a deeper analysis of ways to deal with imbalanced hate speech datasets. Our goal is to investigate whether over- or undersampling is the better choice and at which class ratio. To keep the focus on the individual datasets, we perform our experiments monolingually, testing on the same language as for training. We tune hyperparameters on the corresponding development sets.

We observe from Table 4 that DE-TRAIN not only has a different class ratio than EN-TRAIN but is also much smaller. Therefore to perform as little duplication as possible we select a set of class ratios for sampling that are based around the ratios of these unmodified datasets. The ratios we sample are 7:1 (as in EN-TRAIN), 2:1 (an imbalanced scenario to a lesser degree) and 1:1 (the balanced scenario). The sampled datasets are named with their language code initials appended with either ‘US’ if produced by undersampling or ‘OS’ if produced by oversampling. To match the 7:1 ratio of ‘noHate’ to ‘Hate’ in EN-TRAIN we produce an oversampled version of DE-TRAIN called DE-OS[7:1] with a 7:1 class ratio. Next we produce EN-US[2:1] and DE-US[2:1] by removing appropriate amounts of ‘noHate’ examples from EN-TRAIN and DE-TRAIN, respectively. We create EN-US[1:1] and DE-US[1:1], which were produced by removing ‘noHate’ examples until their number matched the number of ‘Hate’ examples in their respective datasets. Finally, EN-OS[1:1] and DE-OS[1:1] was produced by duplicating the ‘Hate’ examples until they match the number of ‘noHate’ examples. Table 18 shows label statistics of the resulting datasets for

**Table 18:** English and German training datasets used in our monolingual experiments. Sampled datasets were produced from EN-TRAIN and DE-TRAIN respectively. (See Table 4 for data on DE-TRAIN)

	Nohate	Hate	Ratio (approx.)
EN-TRAIN	9018	1281	7:1
EN-US[2:1]	2562	1281	2:1
EN-US[1:1]	1281	1281	1:1
EN-OS[1:1]	9018	9018	1:1
DE-OS[7:1]	5985	855	7:1
DE-US[2:1]	1710	855	2:1
DE-US[1:1]	855	855	1:1
DE-OS[1:1]	3345	3345	1:1

English and German.

The results of our experiments for the CNN, the BiLSTM and mBERT architectures are presented respectively in Tables 19, 20 and 21. The CNN achieved its highest classwise ‘Hate’ scores with EN-OS[1:1] and EN-US[1:1]. Among the German datasets, the CNN achieved its best ‘Hate’ F1 on the two balanced datasets and on DE-US[2:1]. Classwise ‘Hate’ performance on DE-OS[7:1] was significantly lower. In particular, the CNN achieved noticeably lower ‘Hate’ recall on this dataset than on DE-US[2:1] and DE-US[1:1], despite the ‘Hate’ precision scores being similar. Since the total amount of ‘Hate’ samples in these three datasets was the same (see Table 18), the class imbalance of DE-OS[7:1]is the likeliest explanation.

The BiLSTM achieved its highest ‘Hate’ F1 on EN-OS[1:1], and its highest German ‘Hate’ F1 scores on DE-US[1:1] and DE-OS[1:1]. The two German datasets with imbalanced distributions yielded a slightly poorer performance in the ‘Hate’ class, similar to what was observed with the CNN. It is additionally worth noting that although the BiLSTM achieved similar ‘Hate’ F1 scores on DE-US[2:1] and DE-OS[7:1], its ‘noHate’ precision and recall on the latter dataset were lower than those from the former. This indicates that for DE-OS[7:1] the BiLSTM could only achieve good performance in the minority class by overfitting to it. Taken together with our observations from the CNN, this illustrates the detrimental effect of an imbalanced class ratio within small corpora.

mBERT had the best overall performance among the three architectures. Similar to the trend shown by the previous models, it achieved its highest macro-average F1 score on EN-OS[1:1], as well as its highest ‘Hate’ and ‘noHate’ scores. This benefit could have been due to the larger size of EN-OS[1:1] compared to the other corpora. The fact that scores for each class ratio also tended to be higher with the English datasets points to the model’s strength with English training data, despite its multilinguality. Classwise performance on the [7:1] and [2:1] datasets is slightly stronger in the ‘noHate’ class than in ‘Hate’, reflecting the datasets’ skew towards ‘noHate’.

Among the [1:1] datasets, mBERT’s classwise ‘Hate’ scores and macro-average F1 scores tended to be higher for the oversampled versions of a particular language than for the undersampled versions. For example, mBERT achieved a ‘Hate’ F1 of 63.7 on DE-OS[1:1] compared to 62.9 on DE-US[1:1]. The oversampled dataset also yielded better ‘noHate’ recall and F1, as well as better macro-average scores. The same pattern is observed with EN-US[1:1] and EN-OS[1:1], with the latter dataset giving significantly better scores in every category.

**Table 19:** Monolingual CNN performance after training on the various sampled datasets

Trainset	Accuracy	Nohate			Hate			Macro-Avg		
		P	R	F1	P	R	F1	P	R	F1
DE-OS[7:1]	74.48	81.65	86.96	84.22	38.48	29.44	33.36	60.06	58.2	58.79
DE-US[2:1]	71.25	85.38	76.36	80.62	38.21	52.78	44.33	61.8	64.57	62.47
DE-US[1:1]	69.19	85.55	72.98	78.77	36.26	55.5	43.86	60.9	64.24	61.31
DE-OS[1:1]	77.6	83.78	88.54	86.1	47.95	38.13	42.48	65.87	63.33	64.29
EN-TRAIN[7:1]	58.49	77.95	66.00	71.48	19.47	30.58	23.79	48.71	48.29	47.64
EN-US[2:1]	59.02	77.41	67.78	72.27	18.08	26.45	21.48	47.75	47.11	46.88
EN-US[1:1]	78.16	97.06	77.28	86.05	35.33	84.13	49.77	66.2	80.71	67.91
EN-OS[1:1]	87.35	95.97	89.23	92.48	50.54	74.6	60.26	73.25	81.92	76.37

**Table 20:** Monolingual BiLSTM performance after training on the various sampled datasets

Trainset	Accuracy	Nohate			Hate			Macro-Avg		
		P	R	F1	P	R	F1	P	R	F1
DE-OS[7:1]	24.97	74.65	8.26	14.87	20.19	89.22	32.93	47.42	48.74	23.90
DE-US[2:1]	72.59	81.21	84.45	82.8	35.29	30.27	32.59	58.25	57.36	57.7
DE-US[1:1]	61.89	81.87	65.78	72.95	28.21	47.99	35.54	55.04	56.89	54.24
DE-OS[1:1]	72.71	81.81	83.65	82.72	36.57	33.64	35.04	59.19	58.64	58.88
EN-TRAIN[7:1]	67.96	95.61	66.28	78.28	25.77	79.37	38.91	60.69	72.82	58.6
EN-US[2:1]	81.02	92.39	85.25	88.67	34.38	52.38	41.51	63.38	68.81	65.09
EN-US[1:1]	63.88	97.71	59.95	74.31	25.0	90.48	39.18	61.35	75.21	56.74
EN-OS[1:1]	79.59	93.37	82.44	87.56	33.63	60.32	43.18	63.5	71.38	65.37

**Table 21:** Monolingual mBERT performance after training on the various sampled datasets

Trainset	Accuracy	Nohate			Hate			Macro-Avg		
		P	R	F1	P	R	F1	P	R	F1
DE-OS[7:1]	75.00	77.54	87.42	82.19	67.62	50.92	58.09	72.58	69.17	70.14
DE-US[2:1]	73.50	81.14	77.94	79.51	60.28	64.89	62.50	70.71	71.42	71.00
DE-US[1:1]	71.72	82.61	72.36	77.14	56.81	70.47	62.90	69.71	71.41	70.02
DE-OS[1:1]	74.15	81.93	78.03	79.93	61.01	66.64	63.70	71.47	72.33	71.81
EN-TRAIN[7:1]	84.94	86.11	96.44	90.99	76.12	42.15	54.26	81.12	69.3	72.62
EN-US[2:1]	84.06	92.84	86.44	89.53	59.87	75.21	66.67	76.35	80.83	78.10
EN-US[1:1]	78.11	89.93	81.33	85.41	48.78	66.12	56.14	69.35	73.72	70.78
EN-OS[1:1]	99.12	100.00	98.89	99.44	96.03	100.00	97.98	98.02	99.44	98.71

In addition, despite DE-OS[1:1] and EN-OS[1:1] having identical class ratios, mBERT’s much higher scores with the latter training set point to this architecture’s need for a large amount of data. However, the transformer’s significantly higher ‘Hate’ scores show that it is generally better able to cope with smaller dataset sizes than the BiLSTM and CNN. Among the three architectures examined, mBERT was the most successful at maintaining good minority-class performance on our relatively small corpora, making this architecture the better choice for low-resource setups.

Although all three architectures achieved their best English ‘Hate’ F1 scores on the oversampled, balanced EN-OS[1:1], only mBERT had the same success in German with DE-OS[1:1]. The CNN’s German ‘Hate’ F1 was the highest with DE-US[2:1], while the BiLSTM’s was with DE-US[1:1]. This indicates that having a balanced class distribution is not the sole deciding factor for good minority-class performance, at least for small corpora. Among the [1:1] German datasets, the use of oversampling or undersampling did not play a deciding role for ‘Hate’ F1 performance. The difference between the ‘Hate’ F1 scores of EN-OS[1:1] and EN-US[1:1] was much higher, suggesting that oversampling the minority class might be a better option than undersampling the majority if the majority class is significantly larger. Additionally, our experiments indicate that the duplicated examples present in the oversampled datasets did not pose a significant problem for our models. More research will have to be done to confirm these conclusions, as well as to shed light on the exact interplay between class distribution and dataset size on minority class performance.

## Conclusion

Building automatic hate speech detection systems for low-resource languages is difficult due to the small amount of available datasets. Our goal in this paper was to investigate whether cross-lingual transfer learning could be used to mitigate the problem of data scarcity and additionally to highlight the problems related to data annotations: incompatible label definitions and class imbalance. We chose an English dataset with a broad hate speech definition for training and a similar German corpus for testing. Although the datasets were similar, we had to simplify the complex annotation schema of the target language dataset into the binary schema of the source dataset to make them compatible for the cross-lingual experiments. Our results showed that cross-lingual transfer learning is indeed an effective tool for hate speech detection in low-resource languages. Additionally, we assembled two corpora of previously-unseen, unlabeled target language data and applied an ensemble of trained classifiers to them. We showed that fine-tuning on these automatically-labeled examples improved classification performance, particularly within the hate speech class. However, our results also show that models can be sensitive to hyperparameters, thus care has to be taken when selecting them. Additionally we investigated the issue of class imbalance in hate speech datasets. We produce several over- and undersampled datasets based on our English and German corpora, using class ratios that reflect the original datasets’ ratios. We test the efficacy of oversampling compared to undersampling and conclude that both may possess advantages for specific dataset scenarios. Our goal for the future is to apply cross-lingual transfer learning to other language pairs with greater syntactic differences than German and English. In addition, since the differences of labeling schemas across various hate speech datasets could prevent the application of transfer learning methods, we aim to develop a method that can effectively combine datasets with different labeling schemas without the need for label modifications. Finally, since cultural differences become relevant in cross-lingual setups, we aim to examine their effect on model performance more thoroughly.

## Footnotes

1 The data samples in this paper are shown for explanatory purposes and do not represent the views of the authors.

2 https://hatespeechdata.com.

3 https://github.com/yoonkim/CNN_sentence/blob/master.

4 Due to privacy concerns, the authors do not make the DE-NEW dataset public but can provide it individually upon request.

5 The website was crawled using the Python Scrapy library.

## References

[CR1] Artetxe M, Labaka G, Agirre E (2018). A robust self-learning method for fully unsupervised cross-lingual mappings of word embeddings. Proceedings of the 56th Annual meeting of the association for computational linguistics.

[CR2] Basile V, Bosco C, Fersini E, Debora N, Patti V, Pardo FMR, Rosso P, Sanguinetti M (2019). Semeval-2019 task 5: multilingual detection of hate speech against immigrants and women in twitter. 13th international workshop on semantic evaluation.

[CR3] Benítez-Andrades JA, González-Jiménez Á, López-Brea Á, Aveleira-Mata J, Alija-Pérez J-M, García-Ordás MT (2022). Detecting racism and xenophobia using deep learning models on twitter data: Cnn, lstm and bert. PeerJ Comput Sci.

[CR4] Bigoulaeva I, Hangya V, Fraser A (2021). Cross-lingual transfer learning for hate speech detection. Proceedings of the first workshop on language technology for equality, diversity and inclusion.

[CR6] Bojanowski P, Grave E, Joulin A, Mikolov T (2017). Enriching word vectors with subword information. Trans Assoc Comput Linguist.

[CR7] Bojar O, Chatterjee R, Federmann C, Haddow B, Hokamp C, Huck M, Logacheva V, Pecina P (2015). Proceedings of the tenth workshop on statistical machine translation.

[CR8] Bretschneider U, Peters R, Bui T (2017). Detecting offensive statements towards foreigners in social media. Proceedings of the 50th Hawaii international conference on system sciences.

[CR9] Chawla NV, Bowyer KW, Hall LO, Kegelmeyer WP (2002). Smote: synthetic minority over-sampling technique. J Artificial Intelligence Res.

[CR10] Conneau A, Lample G, Ranzato M, Denoyer L, J’egou H (2018). Word translation without parallel data.

[CR11] Conneau A, Lample G, Ranzato M, Denoyer L, Jégou H (2018). Word translation without parallel data. Proceedings of the international conference on learning representations.

[CR12] Davidson T, Warmsley D, Macy M, Weber I (2017). Automated hate speech detection and the problem of offensive language. Proceedings of the 11th International AAAI conference on web and social media.

[CR13] de Gibert O, Perez N, García-Pablos A, Cuadros M (2018). Hate speech dataset from a white supremacy forum. Proceedings of the 2nd workshop on abusive language online (ALW2).

[CR14] De Smedt T, Jaki S (2018). Challenges of automatically detecting offensive language online: participation paper for the germeval shared task 2018 (HaUA). Proceedings of the GermEval 2018 workshop.

[CR15] Devlin J, Chang MW, Lee K, Toutanova K (2019). Pre-training of deep bidirectional transformers for language understanding. Proceedings of the 2019 Conference of the North American chapter of the association for computational linguistics: human language technologies.

[CR16] Fišer D, Huang R, Prabhakaran V, Voigt R, Waseem Z, Wernimont J (2018). Proceedings of the 2nd workshop on abusive language online (ALW2).

[CR17] Fortuna P, Nunes S (2018). A survey on automatic detection of hate speech in text. ACM Comput. Surv..

[CR18] Fortuna P, Soler J, Wanner L (2020). Toxic, hateful, offensive or abusive? what are we really classifying? an empirical analysis of hate speech datasets. Proceedings of the 12th language resources and evaluation conference.

[CR19] Gao L, Huang R (2017). Detecting online hate speech using context aware models.

[CR20] Glavaš G, Karan M, Vulic I (2020). Analyzing and detecting abusive language across domains and languages.

[CR21] Gröndahl T, Pajola L, Juuti M, Conti M, Asokan N (2018). All you need is love evading hate speech detection. Proceedings of the 11th ACM workshop on artificial intelligence and security.

[CR22] Jiang A, Zubiaga A (2021). Cross-lingual capsule network for hate speech detection in social media. Proceedings of the 32nd ACM conference on hypertext and social media.

[CR23] Johnson J, Khoshgoftaar T (2019). Survey on deep learning with class imbalance. J Big Data.

[CR24] Kim Y (2014). Convolutional neural networks for sentence classification. Proceedings of the 2014 conference on empirical methods in natural language processing (EMNLP).

[CR25] Koehn P, Hoang H, Birch A, Callison-Burch C, Federico M, Bertoldi N, Cowan B, Shen W, Moran C, Zens R (2007). Moses: open source toolkit for statistical machine translation. Proceedings of the 45th annual meeting of the acl on interactive poster and demonstration sessions.

[CR26] Kozareva, Z. (2006). Bootstrapping named entity recognition with automatically generated gazetteer lists. In: Student Research Workshop. url: https://www.aclweb.org/anthology/E06-3004

[CR27] Kumar R, Ojha AK, Malmasi S, Zampieri M (2018). Benchmarking aggression identification in social media. Proceedings of the first workshop on trolling, aggression and cyberbullying (TRAC-2018).

[CR28] MacAvaney S, Yao HR, Yang E, Russell K, Goharian N, Frieder O (2019). Hate speech detection: challenges and solutions. PLOS ONE.

[CR29] Madukwe K, Gao X, Xue B (2020). (2020) In data we trust: a critical analysis of hate speech detection datasets. Proceedings of the fourth workshop on online abuse and harms.

[CR30] Majumder P, Patel D, Modha S, Mandl T (2019). Overview of the HASOC track at FIRE 2019: hate speech and offensive content identification in Indo-European languages. Proceedings of the 11th forum for information retrieval evaluation.

[CR31] Mathur P, Sawhney R, Ayyar M, Shah R (2018). Did you offend me? classification of offensive tweets in Hinglish language. Proceedings of the 2nd workshop on abusive language online (ALW2).

[CR32] Mikolov, T., Le, Q.V., & Sutskever, I. (2013a). Exploiting Similarities among Languages for Machine Translation. CoRR abs/1309.4

[CR33] Mikolov T, Chen K, Corrado G, Dean J (2013). Efficient estimation of word representations in vector space. 1st international conference on learning representations.

[CR34] Mohammed R, Rawashdeh J, Abdullah M (2020). Machine learning with oversampling and undersampling techniques: overview study experimental results.

[CR35] Nozza D (2021). Exposing the limits of zero-shot cross-lingual hate speech detection. Proceedings of the 59th annual meeting of the association for computational linguistics and the 11th international joint conference on natural language processing.

[CR36] Pamungkas EW, Basile V, Patti V (2021). Towards multidomain and multilingual abusive language detection: a survey. Personal Ubiquitous Comput.

[CR37] Pamungkas EW, Basile V, Patti V (2021). A joint learning approach with knowledge injection for zero-shot cross-lingual hate speech detection. Info Process Manag.

[CR38] Pelicon A, Shekhar R, Martinc M, Škrlj B, Purver M, Pollak S (2021). Zero-shot cross-lingual content filtering: offensive language and hate speech detection. Proceedings of the EACL hackashop on news media content analysis and automated report generation.

[CR39] Poletto F, Basile V, Sanguinetti M, Bosco C, Patti V (2021). Resources and benchmark corpora for hate speech detection: a systematic review. Lang Resour Eval.

[CR40] Ranasinghe T, Zampieri M (2020). Multilingual offensive language identification with cross-lingual embeddings. Proceedings of the 2020 conference on empirical methods in natural language processing (EMNLP).

[CR41] Roberts ST, Tetreault J, Prabhakaran V, Waseem Z (2019). Proceedings of the third workshop on abusive language online.

[CR42] Ross B, Rist M, Carbonell G, Cabrera B, Kurowsky N, Wojatzki M, Beißwenger M, Wojatzki M, Zesch T (2016). Measuring the reliability of hate speech annotations: the case of the European refugee crisis. Proceedings of NLP4CMC III: 3rd workshop on natural languageprocessing for computer-mediated communication.

[CR43] Ruppenhofer J, Siegel M, Wiegand M (2018). Proceedings of the GermEval 2018 workshop.

[CR44] Schmidt A, Wiegand M (2017). A survey on hate speech detection using natural language processing. Proceedings of the Fifth International workshop on natural language processing for social media.

[CR45] Stappen L, Brunn F, Schuller B (2020). Cross-lingual zero- and few-shot hate speech detection utilising frozen transformer language models and axel.

[CR46] Struß J, Siegel M, Ruppenhofer J, Wiegand M, Klenner M (2019). Overview of germeval task 2, 2019 shared task on the identification of offensive language.

[CR47] Vidgen B, Derczynski L (2020). Directions in abusive language training data, a systematic review: garbage in, garbage out. PLOS ONE.

[CR48] Wang X, Wu Z, Lian L, Yu SX (2022). Debiased learning from naturally imbalanced pseudo-labels. CVF Conference on Computer Vision and Pattern Recognition.

[CR51] Waseem Z, Hovy D (2016). Hateful symbols or hateful people? predictive features for hate speech detection on twitter. Proceedings of the NAACL student research workshop.

[CR50] Waseem Z, Chung WHK, Hovy D, Tetreault J (2017). Proceedings of the first workshop on abusive language online.

[CR49] Waseem, Z., Davidson, T., Warmsley, D., Weber, I. (2017b). Understanding abuse: A typology of abusive language detection subtasks. arXiv preprint arXiv:1705.09899

[CR52] Wei C, Sohn K, Mellina C, Yuille A, Yang F (2021). A class-rebalancing self-training framework for imbalanced semi-supervised learning. Proceedings of the IEEE/CVF conference on computer vision and pattern recognition.

[CR53] Wiedemann G, Ruppert E, Jindal R, Biemann C (2018). Transfer learning from LDA to BiLSTM-CNN for offensive language detection in twitter. Proceedings of the GermEval 2018 workshop.

[CR54] Wiegand M, Amann A, Anikina T, Azoidou A, Borisenkov A, Kolmorgen K, Kröger I, Schäfer C (2018). Saarland University’s Participation in the GermEval Task 2018 (UdSW)-examining different types of classifiers and features. Proceedings of the GermEval 2018 workshop.

[CR55] Wiegand M, Siegel M, Ruppenhofer J (2018). Overview of the germeval 2018 shared task on the identification of offensive language. Proceedings of GermEval 2018, 14th conference on natural language processing (KONVENS 2018).

[CR56] Wulczyn E, Thain N, Dixon L (2017). Ex machina: personal attacks seen at scale. Proceedings of the 26th international conference on world wide web.

[CR57] Xi J, Spranger M, Labudde D (2018). CNN-based offensive language detection. Proceedings of the GermEval 2018 workshop.

[CR58] Zampieri M, Malmasi S, Nakov P, Rosenthal S, Farra N, Kumar R (2019). Predicting the type and target of offensive posts in social media. Proceedings of the 2019 Conference of the North American chapter of the association for computational linguistics: human language technologies.

[CR59] Zampieri M, Nakov P, Rosenthal S, Atanasova P, Karadzhov G, Mubarak H, Derczynski L, Pitenis Z, Çöltekin Ç (2020). 2020) Semeval-2020 task 12: Multilingual offensive language identification in social media (offenseval 2020. Proceedings of the fourteenth workshop on semantic evaluation.

[CR60] Zia HB, Castro I, Zubiaga A, Tyson G (2022). Improving zero-shot cross-lingual hate speech detection with pseudo-label fine-tuning of transformer language models. Proceedings of the International AAAI conference on web and social media.

